# Short-term outcomes after major lower limb amputation: meta-analysis

**DOI:** 10.1093/bjsopen/zrag122

**Published:** 2026-10-23

**Authors:** Nick Moody, Rhiannon Nielsen, Megan Lyons, Leanne Murphy, Becky Sandford, David C Bosanquet, Kathryn Chu, Matthew Popplewell, Justine Davies

**Affiliations:** Department of Applied Health Sciences, College of Medicine and Health, University of Birmingham, Birmingham, UK; Gwent Vascular Institute, Royal Gwent Hospital, South East Wales Vascular Network, Newport, Wales, UK; Department of Vascular Surgery, Imperial College NHS Trust, London, UK; Department of General Surgery, Lewisham and Greenwich NHS Trust, London, UK; Department of Vascular Surgery, Guy’s and St Thomas’ NHS Trust, London, UK; Faculty of Life Sciences and Medicine, King’s College London, London, UK; South East Wales Vascular Network, University Hospital of Wales, Cardiff, Wales; Division of Population Medicine, Cardiff University, Cardiff, UK; Centre for Global Surgery, Stellenbosch University Faculty of Medicine and Health Sciences, Cape Town, South Africa; Department of Applied Health Sciences, College of Medicine and Health, University of Birmingham, Birmingham, UK; Department of Applied Health Sciences, College of Medicine and Health, University of Birmingham, Birmingham, UK

## Abstract

**Introduction:**

Mortality after major lower limb amputation is a key factor in the decision to perform surgery. This review synthesizes the evidence on in-hospital and 30-day postoperative mortality after major amputation.

**Methods:**

Electronic databases Medline, Embase, Scopus and African Journals Online were searched using keywords and thesaurus terms in October 2024 and updated in November 2025. Studies published globally and reporting in-hospital or 30-day mortality after major lower limb amputation (defined as at- or above-ankle amputation, or transtibial amputation (TTA) and/or transfemoral amputation (TFA)) and published between 2008 and 2025 were included. Only population level studies were included. Meta-analysis was performed and prediction intervals used to report findings primarily. The Newcastle–Ottawa tool was used to assess the methodological quality of studies.

**Results:**

Fifty-two studies were included (1 067 616 patients). Only three studies were from low- and middle-income countries (two from Brazil, one from Togo). Most studies were either from North America (42%) or Europe and Central Asia (36%). Twenty-six studies reported in-hospital mortality and 26 reported 30-day mortality. Prediction intervals were 3.6–24.5% (point estimate 10.0%, 95% confidence interval 8.2% to 12.1%; 664 608), and 4.0–20.4% (point estimate 9.4%, 8.0% to 11.0%; 390 030) for in-hospital and 30-day mortality, respectively. In-hospital mortality prediction intervals were 2.9–12.7% (point estimate 6.2%, 5.1% to 7.7%; 77 204) for TTAs and 5.6–23.3% (point estimate: 11.8%, 9.6% to 14.4%; 78 632) for TFAs. Prediction intervals for 30-day mortality were 2.5–11.6% (point estimate 5.5%, 4.5% to 6.7%; 87 971) for TTAs and 4–26.6% (point estimate 10.9%, 8.3% to 14.2%; 42 145) for TFAs. Heterogeneity was high (> 75%) across all analyses.

**Conclusion:**

Most studies reporting short-term outcomes after major lower limb amputation are from high-income countries. Reported mortality is highly variable, but prediction intervals can be used to estimate mortality in future studies and inform shared decision-making.

## Teaser

This review summarizes the short-term in-hospital and 30-day outcomes after major lower limb amputation. It demonstrates the variability in published estimates of mortality after amputation, and the use of prediction intervals to understand these estimates in a relevant and comprehensible way.

## Introduction

The decision to perform a major lower limb amputation (herein referred to as major amputation) is often predicated on the assumption that it will result in improved life expectancy and/or improved quality of life^1,2^. Despite this, postoperative mortality remains a significant risk^3,4^. This risk may be the result of the inherent morbidity associated with those who undergo amputations, who often have underlying diabetes mellitus (DM), cardiovascular disease, and frailty^5,6^. It may also be the result of delays in preoperative or operative intervention, or failure to rescue after surgery in those who develop complications^4,7,8^.

Measures to reduce postoperative mortality and morbidity have been increasingly adopted in recent years, with a focus on reducing the rate of surgical site infections, systemic infections, and cardiovascular complications^9,10^. Meanwhile, there has been an increasing focus on prediction tools, which aim to improve the ability to understand who is likely to benefit from an amputation^10–12^. In low- and middle-income countries (LMICs), the importance of postoperative mortality, as one of the six key indicators of the strength of a country’s surgical system, has been highlighted by the Lancet Commission on Global Surgery^13–15^.

Understanding the rate of postoperative complications and mortality is important for clinicians, patients, researchers, and policy makers globally. It facilitates shared decision-making with patients, whilst also providing a benchmark on local, regional, and national levels. It can also inform the design of studies that examine postoperative outcomes after amputation by facilitating power calculations. This is particularly relevant in geographical regions where there may be little published evidence on outcomes after amputation.

The aim of this systematic review is to investigate the in-hospital (regardless of length of stay in hospital) and 30-day postoperative mortality after major amputation in population-based or regional studies. This is reported using prediction intervals to demonstrate the likely mortality estimates based on published data. In doing so, this review aims not only to synthesize the heterogenous evidence but also highlight gaps in the data on outcomes after major amputation.

## Methods

This review followed the PRISMA guidelines for systematic reviews (PRISMA checklist, *Table S1*)^16^. This study reports a predefined analysis of a larger systematic review and meta-analysis (PROSPERO: CRD42024581913)^17^.

### Search strategy

Searches were initially performed in October 2024 and re-run in November 2025. Electronic databases Medline, Embase, Scopus and African Journals Online were searched using keywords and thesaurus terms. The search was developed in Medline and then adapted for other databases (for full search strategy, see *Table S2*). Grey literature (non-peer-reviewed articles) was identified using www.base-search.net, the African Digital Research Repositories, and a customized Google search using the search terms ‘amputation’ OR ‘major amputation’ and ‘outcomes’ OR ‘death’ OR ‘Mortality’. The reference lists of relevant articles were reviewed to identify further relevant studies.

### Inclusion criteria

Patients who underwent major lower limb amputation, regardless of cause, were included. Major amputation was defined as any amputation at or above the level of the ankle. Studies were included if they reported outcomes in a population with any major amputation, or in those who underwent transfemoral amputations (TFAs) and/or transtibial amputations (TTAs). Studies were included if they reported mortality outcomes in hospital (that is, in the period of hospitalization immediately after the operation, including if the period was greater than 30 days) or at 30 days (that is, within 30 days of the amputation date), either as a primary or a secondary outcome. Only population-level studies were included, which were those across broad and well defined regional or national populations^18^. Observational studies (prospective and retrospective) and regional, national, and international registry reports were included. Studies were excluded if they combined mortality outcome data of major and minor amputations (distal to ankle joint) or reported in preselected population subgroups, such as only those who underwent revascularization operations or those on renal dialysis. This was to avoid bias, as such studies may not be reflective of outcomes in the general population who undergo major amputation. Studies that were primarily war/conflict based were also excluded. To ensure contemporary relevance, studies published from 2008 onwards were included (the year of publication of the World Health Organization action plan for the global strategy for the prevention and control of noncommunicable diseases)^19^. Detailed inclusion and exclusion criteria and PICO framework are detailed in *Table S3*.

### Screening and extraction

Identified reports were imported into Covidence (Veritas Health Innovation, Melbourne, Australia) and duplicates removed. All titles and abstracts were screened against the inclusion and exclusion criteria by two reviewers independently (two of N.M., L.M., M.L., R.N.). Once screening was complete, disagreements were resolved by discussion between the two reviewers. Disagreements that could not be resolved were referred to a third reviewer (J.D.). Of the selected reports, two reviewers (of N.M., L.M., A.W.) independently screened the full texts with exclusion reasons recorded. Disagreements were once again resolved by discussion. Data extraction was performed by two reviewers (N.M. and L.M.) who initially independently extracted data from selected batches of 25 studies and assessed agreement. Once agreement was over 90%, full data extraction was performed independently, with 10% of studies randomly selected to ensure agreement remained over 90% at pre-agreed points. Disagreements were resolved by discussion. Data extracted included general study details, country of study, socioeconomic status, geographical region, number of patients included, mean age, sex distribution, DM prevalence, proportion of TFA *versus* TTA, and mortality outcomes. If mortality outcomes of TTA and TFA were provided separately, they were also extracted. World Bank classifications of LMICs and high-income countries were used to classify socioeconomic status of countries where the study originated at time of publication, and World Bank regions were used to classify regions from which studies originated. If two or more studies used identical data over the same time period, the most recent study was used.

### Quality assessment

The Newcastle–Ottawa tool (NOS) was used to assess the methodological quality of the studies^20^. Certainty assessments for each of the meta-analysed results were performed using the Grading of Recommendations Assessment, Development and Evaluation (GRADE) tool^21^.

### Data synthesis

Analysis was performed for in-hospital and 30-day mortality. Meta-analysis of proportions was performed using a random-effects model with restricted maximum likelihood (REML). Proportions were logit-transformed before analysis. Confidence intervals (c.i.) were calculated using the Hartung–Knapp method. Subgroup analysis of in-hospital and 30-day mortality of TFAs and TTAs was also performed due to the well documented difference in mortality after each respective operation. Statistical heterogeneity was assessed using the *I*^2^ statistic. To manage heterogeneity and inform future studies, prediction intervals are reported as well as point estimates and 95% c.i. Prediction intervals account for between-study heterogeneity and represent the plausible range of mortality outcomes in a new study^22^ drawn from the same underlying distribution. By taking heterogeneity into account, they provide a wider estimate than 95% c.i. Forest plots are used to display study estimates, with pooled random-effects estimates and 95% prediction intervals.

In addition, univariable random-effects meta-regression using REML was performed. This examined *a priori* identified and extracted study-level covariables: geographical region (defined by the World Bank), income level of country of study at the time of publication (defined by the World Bank), year of study publication, mean age of population, percentage of amputations that were TFA, and DM prevalence in the study population. Univariable meta-regression was performed for all covariables. If positive univariable associations were identified, these were explored by performing tests of publication bias relevant to the variables being explored. Multivariable meta-regression was not performed due to inconsistent reporting of covariables across studies, making multivariable models underpowered. Leave-one-out analysis was performed to assess for large single-study effects. Presence of publication bias was explored for the meta-analysed subgroups that included ten or more studies, using Egger’s regression and visualization of funnel plots, and a two-sided *P* value of < 0.050 was considered statistically significant. Meta-analyses were performed using Stata^®^ (version 18.5, StataCorp LLC, College Station, TX, USA).

## Results

### Search results

Titles and abstracts of 13 948 peer-reviewed reports and 1461 grey literature reports were screened (*Fig. 1*). Fifty-two studies were included (total population 1 067 616).

**Figure 1:**
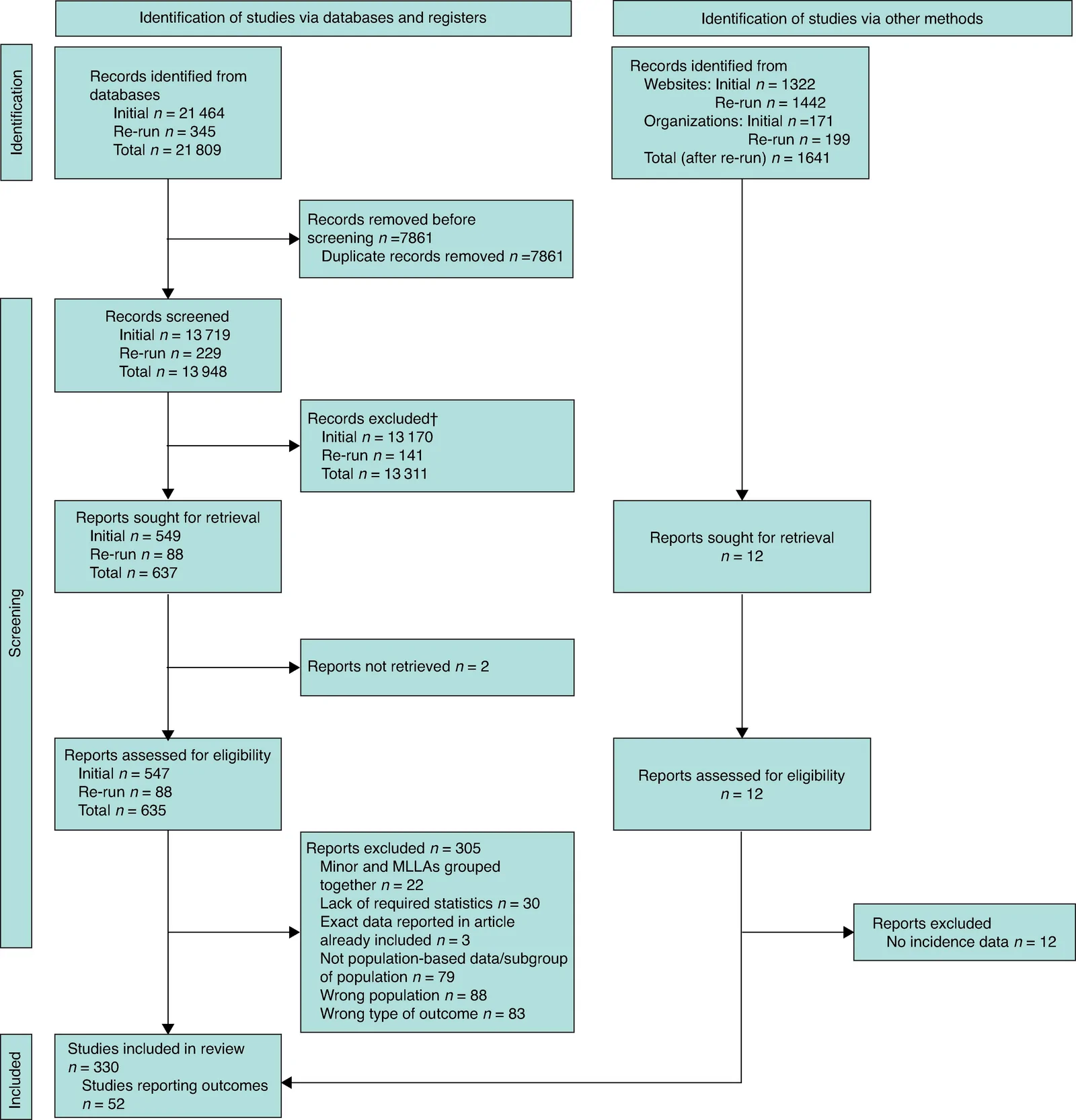
PRISMA 2020 flow diagram showing screening process *Initial and re-run searches were performed approximately one year apart. For websites and organisations, re-run values are cumulative totals at the time of re-run rather than additional records. MLLA, major lower limb amputation.

Forty-eight studies^8,11,23–68^ (1 038 298) reported the outcomes of all major lower limb amputations, and four studies^69–72^ (29 318) reported only outcomes after TTA. In-hospital mortality was reported by 26 studies^8,27,30,31,33,48–68^ (664 608), with study details in *Table 1* (population *n* is derived from 25 of 26 studies as 1 study^49^ did not provide a population denominator). Thirty-day mortality was reported by 26 studies^11,23–47^ (390 030), with study details in *Table 2* (4 studies reported both in-hospital and 30-day mortality)^27,30,31,33^.

**Table 1:** In-hospital mortality, study characteristics and reported findings

Reference	Inclusion (any or first amputation)*	Study exclusion criteria†	Study period (years)	Population (*n*)	Age (mean)	Male (%)	Diabetes prevalence (%)	Transfemoral amputations (%)	In-hospital mortality (%)‡
Portela *et al.*, 2024 (Brazil)^61^	Any	Malignancy	2008–2020 (13)	280 942	-	-	-		12.0
Biagioni *et al.*, 2022 (Brazil, Sao Paolo)^50^	Any	Malignancy	2009–2020 (12)	59 582	-	-	-		14.2
Waton *et al.*, 2024 (UK)^67^§	Any	Trauma	2023 (1)	3688	-	74.4	61.8	46.0	7.0
Waton *et al.*, 2025 (UK)^68^§	Any	Trauma	2024 (1)	3877	-	74.4	62.7	45.9	7.1
Waton *et al.*, 2023 (UK)^66^§	Any	Trauma	2022 (1)	3505	-	74.0	61.0	47.2	7.8
Waton *et al.*, 2022 (UK)^63^§	Any	Trauma	2021 (1)	3068	-	-	-	50.0	7.6
Waton *et al.*, 2021 (UK)^64^§	Any	Trauma	2019–2020 (2)	6429	-	-	-	49.8	7.1
Waton *et al.*, 2019 (UK)^65^§	Any	Trauma	2016–2018 (3)	9508	-	-	-	47.6	7.7
Ambler *et al.*, 2020 (UK)^48^	Any	Trauma	2014–2016 (3)	9549	70.0	70.5	53.0	47.3	9.1
Maheswaran *et al.*, 2024 (England)^58^	Any	Trauma, malignancy	2006–2018 (13)	47 249	-	-	48.0	50.7	14.9
Moxey *et al.*, 2012 (England)^8^	Any	Trauma, malignancy	2002–2006 (4)	14 168	70.0	65.9	43.7	50.0	17.0
Moxey *et al.*, 2010 (England)^60^	Any	-	2003–2008 (7)	25 578	-	-	39.4	50.2	16.7
Fortington *et al.*, 2013 (Netherlands, Northern province)^33^#	First	Trauma, cancer CRPS, congenital	2003–2004 (2)	299	74.0	60.0	50.0	43.5	19.0
Malyar *et al*., 2013 (Germany)^59^	Any	Non-PAD patients	2005–2009 (5)	51 819	-	-	-		19.0
Lopez-de-Andres *et al*., 2016 (Spain)^56^	Any	Trauma	2003–2013 (11)	64 710	73.7	64.2	-		11.4
Feinglass *et al*., 2008 (USA, Illinois)^54^	Any	Trauma, malignancy, aged < 35	1987–2004 (18)	28 042	72.2	50.0	58.6	52.8	8.2
Humphries *et al.*, 2016 (USA, California)^55^	First in PAD or DM	Trauma, malignancy, no PAD or DM	2005–2011 (7)	11 896	69.0	62.0	64.0		23.0
Davenport *et al.*, 2012 (USA)^30^#	Any with DM or PAD	Pre-op DNR, ASA 5, emergency op, malignancy	2005–2009 (5)	6188	67.4	60.9	-	37.7	3.4
Easterlin *et al.*, 2013 (USA)^31^#	Any	DNR, malignancy	2005–2009 (5)	9244	66.0	62.0	62.0		5.0
Endoh *et al.*, 2017 (Japan)^53^	Any	Bilateral, malignant	2010–2014 (5)	11 626	72.4	63.1	-	51.6	11.6
Tchankoni *et al.*, 2024 (Togo)^62^	Any	Trauma	2016–2021(6)	322	60.0	58.1	54.0	36.0	9.0
Birmpili *et al.*, 2024 (Multicountry)^27^#	Any	-	2020 (0.25)	609	65.0	76.8	80.6	-	11.0
Behrendt *et al.*, 2018 (Multicountry)^49^¶	Any	Non-PAD	2010–2014	-	-	-	-	-	Germany 18.5 UK 7.7 Hungary 20.9 Sweden 14.5 Austria 14.3 Finland 6.1 Norway 6.3
Di Giovanni *et al.*, 2021(Italy, Abruzzo region)^52^	Any, DM-related	Traumatic, malignancy, non-DM	2006–2015 (10)	579	60.6		100.0		8.5
CIHI, 2024 (Canada)^51^	First, DM-related	Non-DM	2020–2023 (4)	9252	-	72.8	100.0		8.2
Low *et al.*, 2017 (USA)^57^	Any, trauma related	Non-traumatic	2011–2012 (2)	2879	42.3	80.4	-	37.5	6.2

^*^Inclusion criteria refers to who was included in study, ‘any’ refers to whether any amputation was included, and ‘first’ refers to whether only a person’s first amputation was included. - Indicates that information was not available. †Non-DM or non-PAD in exclusion criteria refers to the exclusion of those with non-DM or non-PAD related major amputation, respectively. ‡In-hospital mortality was not censored at 30 days and therefore mortality rates include those reported as in-hospital mortality after 30 days. §Studies by Waton *et al.* are annual UK National vascular registry reports. ¶Behrendt *et al.*^49^ did not provide a population denominator value and was not included in meta-analysis, but did provide mortality rates by country. #Easterlin *et al.*^31^, Birmpili *et al.*^27^, Fortington *et al.*^33^, and Davenport *et al.*^30^ reported both 30-day and in-hospital mortality. CRPS, complex regional pain syndrome; PAD, peripheral arterial disease; DM, diabetes mellitus; DNR, exclusion of patients with a do-not-resuscitate order; ASA, American Society of Anesthiologists; CIHI, Canadian institute of health information.

**Table 2:** Thirty-day mortality, study characteristics, and reported findings

Reference	Inclusion (any or first amputation)*	Study exclusion criteria†‡	Study period (years)	Population (*n*)	Age (mean)	Male (%)	Diabetes prevalence (%)	Transfemoral amputations (%)	30-day mortality (%)
Vamos *et al.*, 2010 (England)^46^	Any	Trauma	2004–2005 (2)	4424	76.4	-	-		12.7
Fortington *et al.*, 2013 (Netherlands, northern region)^33^	First	Trauma, cancer CRPS, congenital	2003–2004 (2)	299	74.0	60.0	50.0	43.0	22.0
Brix *et al.*, 2024 (Denmark)^28^	First	Aged < 50, trauma, malignancy	2010–2021 (12)	11 205	74.4	62.5	48.5	65.0	18.8
Fard *et al.*, 2020 (Netherlands, Northern Region)^73^	Any	Trauma, malignancy, CRPS, non-DM infection	2012–2013 (2)	382	72.0	65.0	56.0	31.2	14.0
Easterlin *et al.*, 2013 (USA)^31^	Any	DNR, malignancy	2005–2009 (5)	9244	66.0	62.0	62.0		8.1
Tan *et al.*, 2015 (USA)^44^	Any	Intraop event	2005–2011 (7)	12 464	-	60.8	60.4	46.0	8.8
Iannuzzi *et al.*, 2013 (USA)^38^	Any	-	2005–2010 (6)	11 038	-	61.0	-	42.9	8.9
Gabel *et al.*, 2018 (USA)^34^	Any	TKA	2013–2015 (3)	2939	66.0	64.0	67.0	56.3	5.0
Andersen *et al.*, 2021 (USA)^24^	Any		2012–2017 (6)	3440	66.0	65.0	67.0	40.8	3.8
Karam *et al.*, 2013 (USA)^42^	Any		2005–2008 (4)	6839	62.0	61.0	68.0	41.4	9.1
Nelson *et al.*, 2012 (USA)^43^	Any		2005–2009 (5)	9368	67.8	60.7	60.4	43.1	9.2
Andersen *et al.*, 2019 (USA)^23^	Any	Bilateral same day amputation	2012–2017 (6)	6701	65.5	64.5	68.2	42.5	6.8
Tse *et al.*, 2021 (USA)^45^	Any	Trauma	1999–2018 (10)	47 197	66.4	98.9	62.6	57.4	11.2
Beeson *et al.*, 2023 (USA, California)^26^	Any, PAD diagnosis	Trauma, patients without PAD	2007–2018 (12)	26 699	78.0	67.0	-	33.0	4.8
Hasanadka *et al.*, 2011 (USA)^37^	Any, PAD diagnosis	Non-PAD patients, aged < 50	2005–2008 (3)	4250	-	59.3	62.0	45.7	9.6
Davenport *et al.*, 2012 (USA)^30^	Any with DM or PAD	Preop DNR, ASA 5, emergency op, malignancy	2005–2009 (5)	6188	67.4	60.9	-	37.3	7.6
Jones *et al.*, 2012 (USA)^41^	First, PAD diagnosis	Non-PAD-related	2000–2008 (9)	186 338	78.5	48.1	60.3		13.5
Yamada *et al.*, 2016 (Japan)^47^	First	Trauma, non-DM infection, malignancy	2007–2012 (6)	8565	73.2	63.5	44.0	52.0	6.4
JSVS, 2025 (Japan)^40^	Any	-	2019 (1)	583	-	-	-	58.5	7.9
Hart *et al.*, 2024 (New Zealand)^36^	Any	Trauma, malignancy	2010–2021 (12)	4242	70.0 (median)	63.7	69.8		9.7
Birmpili *et al.*, 2024 (Multicountry)^27^	Any	-	2020 (0.33)	609	65.0	76.8	80.6		12.3
Gwilym *et al.*, 2022 (Multicountry)^11^	Any	Trauma, malignancy	2020–2021 (7 months)	523	68.0	80.0	62.9		9.7
Aziz *et al.*, 2020 (Austria)^25^	First, DM-related	Non-DM	2014–2017 (4)	2165	73.0	62.9	100.0	56.5	13.5
Gurney *et al.*, 2018 (New Zealand)^35^	Any, DM related	Non-DM	2005–2014 (10)	2570	68.7	65.2	100.0		11.1
Chahrour *et al.*, 2022 (USA)^29^	First, DM-related	Non-DM	2005–2017 (8)	21 499	66.0	64.3	100.0	36.5	8.0
Icks *et al.*, 2011 (Germany)^39^	First, DM-related	Non-DM	2005–2007 (3)	259	69.3	75.7	100.0%	38.0	10.0

^*^Inclusion criteria refers to who was included in study, ‘any’ refers to whether any amputation was included, and ‘first’ refers to whether only a person’s first amputation was included. - Indicates that information was not available. †Non-DM or non-PAD in exclusion criteria refers to the exclusion of those with non-DM or non-PAD related major amputation, respectively. ‡Trauma in exclusion criteria refers to the exclusion of those with trauma-related major amputation. CRPS, complex regional pain syndrome; DM, diabetes mellitus; DNR, exclusion of patients with a do-not-resuscitate order; Intraop event, intraoperative adverse event; TKA, through-knee amputation; PAD, peripheral arterial disease; Preop, preoperative; ASA, American Society of Anesthiologists; JSVS, Japanese Society of Vascular Surgery.

Of the included 52 studies, 22 (42%) were from North America^23,24,26,29–31,34,37,38,41–45,51,54,55,57,69–72^, 19 (37%) were from Europe and Central Asia^8,25,28,32,33,46,48,52,56,58–60,63–68^ and 5 (10%) were from East Asia and the Pacific^35,36,40,47,53^. Three of 52 studies were from LMICs; 1 from Togo^62^ and 2 from Brazil^50,61^. Seven of 52 (14%) were regional or subnational studies^26,33,50,52,54,55,73^, 3 were multicountry studies^11,27,49^, and the rest were national. Mean age of the study population was reported by 31 studies (median 68.9, interquartile range (i.q.r.) 66.0–72.9); the remainder did not report mean age or only reported age groups. Thirty-three (64%) studies reported the prevalence of DM in their study population (median 62.0%, i.q.r. 56.0–68.2). Thirty-three (64%) studies reported the percentage of all amputations that were TFAs (median 46.0%, i.q.r. 41.4–50.7). Thirty-six studies reported sex distribution, with the reported median male population 64.1% (i.q.r. 61.0–71.1). Details on studies reporting these data are in *Tables 1*, *2*.

### Quality assessment

Most studies were retrospective in nature. Forty of 52 studies (77%) achieved a NOS of 7 or more, suggesting good study quality. Full details of NOS are in *Table S4*. One study reporting in-hospital mortality was not included in the meta-analysis as it did not provide a figure for the number of people undergoing amputation, only percentage outcome figures^49^. GRADE certainty assessments are provided in *Table 3* and further detailed in *Table S5*.

**Table 3:** Meta-analysis outcomes: intervals and point estimates of in-hospital and 30-day outcomes after amputation with GRADE certainty assessment and Egger’s regression *P* value to indicate impact of small study effects

	95% prediction interval (%)	Point estimate %*	No. of studies (population, *n*)	GRADE	Egger’s regression *P*
**In-hospital mortality**					
All amputations	3.6, 24.5	10.0 (8.2, 12.1)	25 (664 608)	⨁⨁◯◯ Low	0.239
Transfemoral amputation	5.6, 23.3	11.8 (9.6, 14.4)	12 (78 632)	⨁◯◯◯ Very low	< 0.001
Transtibial amputation	2.9, 12.7	6.2 (5.1, 7.7)	12 (77 204)	⨁◯◯◯ Very low	< 0.001
**Thirty-day mortality**					
All amputations	4.0, 20.4	9.4 (8.0, 11.0)	26 (390 030)	⨁◯◯◯ Very low	0.107
Transfemoral amputation	4.0, 26.6	10.9 (8.3, 14.2)	12 (42 145)	⨁◯◯◯ Very low	0.044
Transtibial amputation	2.5, 11.6	5.5 (4.5, 6.7)	16 (87 971)	⨁⨁◯◯ Low	0.822

^*^Values in parentheses are 95% confidence intervals. GRADE, Grading of Recommendations Assessment, Development, and Evaluation.

### In-hospital mortality

Of the 26 studies reporting in-hospital mortality, 10 were from the UK^8,48,58,60,63–68^ (6 were annual registry reports from the UK National Vascular Registry (NVR), whilst 1^48^ study used data based on the NVR (which used NVR data from 2014–2016, therefore NVR reports covering this period were excluded)). Four further studies were from Europe and Central Asia^33,52,56,59^, six from North America^30,31,51,54,55,57^, and one from Japan^53^. One study^49^ used multicountry vascular registry data to report mortality outcomes from a range of European countries. It demonstrated mortality ranging between 6.12% in Finland and 20.88% in Hungary. All three studies^50,61,62^ from LMICs included in this review reported in-hospital mortality. In Togo, reported in-hospital mortality was 9%^62^, and in Brazil it was 12–14.2%^50,61^.

The number of individuals undergoing amputation included in each study ranged between 299^33^ and 280 942^61^. In-hospital mortality ranged between 3.4%^30^ and 23%^55^.

The prediction interval for in-hospital mortality was 3.6–24.5% (point estimate 10%, 95% c.i. 8.2 to 12.1%; *I*^2^ 99.84%, GRADE certainty analysis—low, forest plot: *Fig. 2*).

**Figure 2:**
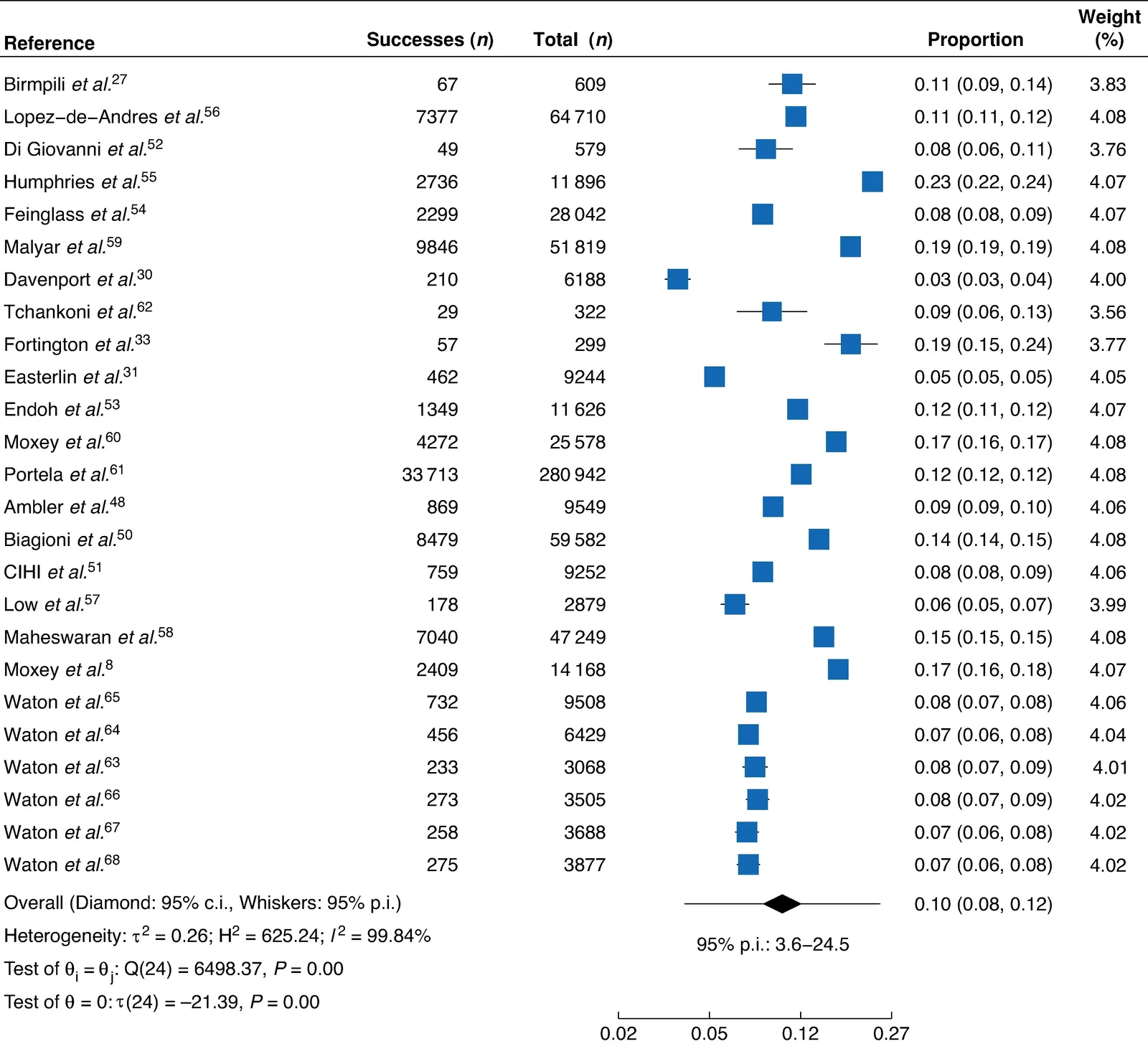
In-hospital mortality after major amputation Whiskers around pooled estimate demonstrates 95% p.i. Waton refers to annual UK National Vascular Registry report. CIHI, Canadian Institute of Health Information. c.i., confidence interval; p.i., prediction interval.

Leave-one-out analysis did not demonstrate a significant impact of any single study on prediction intervals, point estimates, or heterogeneity (*Fig. S1*). Univariable meta-regression did not demonstrate statistically significant association with mortality for the covariables: geographical region (25 studies), DM prevalence (15 studies), year of publication of study (25 studies), percentage who underwent TFA (16 studies), mean age of population (13 studies) or country income status (25 studies) (*Fig. S2*, *Appendix S1*). There was no strong evidence of publication bias (Egger’s regression *P* = .0.239, funnel plot: *Fig. S3*).

#### Sub-group analysis

In-hospital mortality after TFA and TTA was reported by 12 studies^30,48,53,54,58,60,63–68^ (78 632 and 77 204 for TFA and TTA respectively; *Table 4*). The prediction interval for mortality after TTA was 2.9–12.7% (point estimate 6.2%, 95% c.i. 5.1 to 7.7%; *I*^2^ 97.86%, GRADE certainty analysis—very low, forest plot: *Fig. 3*). For TFAs prediction interval was 5.6–23.3% (point estimate 11.8, 9.6 to 14.4%; *I*^2^ 98.90%, GRADE certainty analysis—very low, forest plot: *Fig. 4*). Small study effects were identified in both groups (Egger’s regression value *P* < 0.001 in both TTA and TFA analyses, funnel plot: *Figs. S4*, *S5*, respectively). Leave-one-out analysis did not demonstrate a significant impact of any one individual study in either analysis (*Figs. S6*, *S7*).

**Figure 3:**
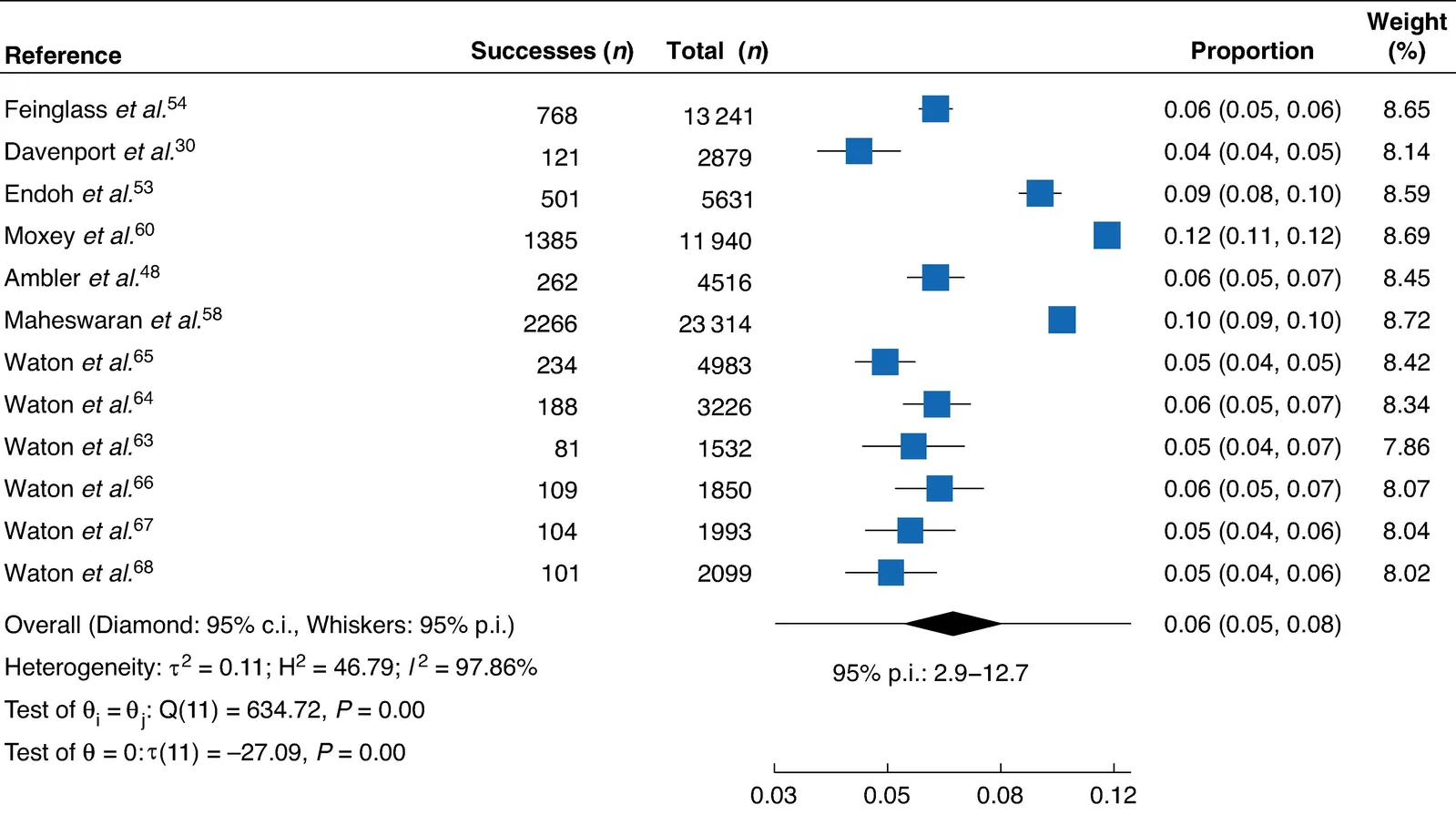
In-hospital mortality after transtibial amputation Whiskers around pooled estimate demonstrates 95% p.i. Waton refers to annual UK National Vascular Registry report. c.i., confidence interval; p.i., prediction interval.

**Figure 4:**
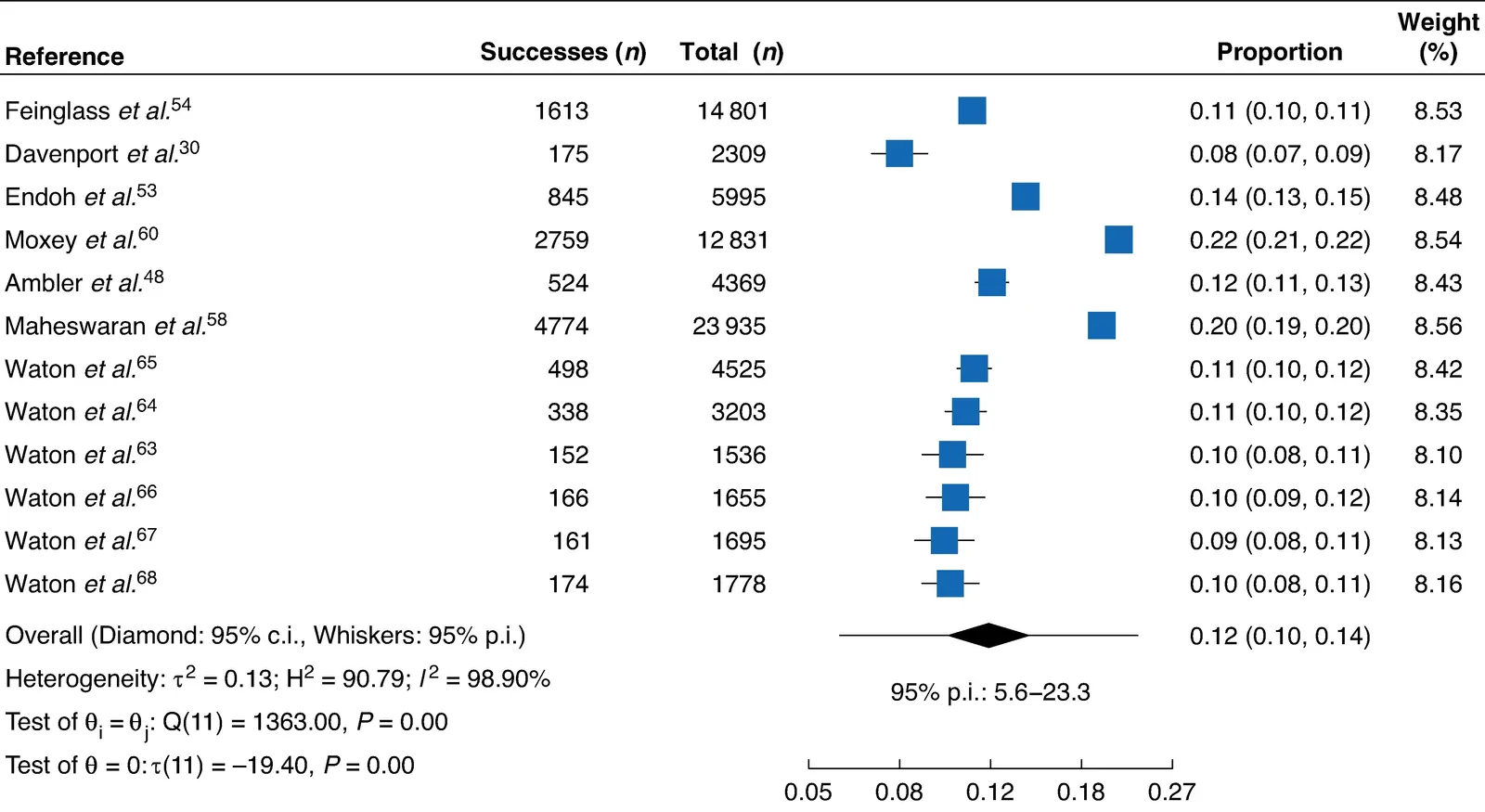
In-hospital mortality after transfemoral amputation Whiskers around pooled estimate demonstrates 95% p.i. Waton refers to annual UK National Vascular Registry report. c.i., confidence interval; p.i., prediction interval.

**Table 4:** Transtibial and transfemoral amputation: in-hospital mortality outcomes in studies that report them stratified by anatomical amputation level

Reference	Country (region)	Transtibial amputation (*n*)	Transtibial amputation in-hospital mortality (%)	Transfemoral amputation (*n*)	Transfemoral amputation in-hospital mortality (%)
Waton *et al.* 2024^67^*	UK	1993	5.2	1695	9.5
Waton *et al.* 2025^68^*	UK	2099	4.8	1778	9.8
Waton *et al.* 2023^66^*	UK	1850	5.9	1655	10.0
Waton *et al.* 2022^63^*	UK	1532	5.3	1536	9.9
Waton *et al.* 2021^64^*	UK	3226	5.8	3203	10.6
Waton *et al.* 2019^65^*	UK	4983	4.7	4525	11.0
Ambler *et al.* 2020^48^	UK	4516	5.8	4369	12.0
Maheswaran *et al.* 2024^58^	England	23 314	10.0	23 935	20.0
Moxey *et al.* 2010^60^	England	11 940	11.6	12 831	21.5
Feinglass *et al.* 2008^54^	USA (Illinois)	13 241	5.8	14 801	10.9
Davenport *et al.* 2012^30^	USA	2879	4.2	2309	7.6
Endoh *et al.* 2017^53^	Japan	5631	8.9	5995	14.1

Values are *n* unless otherwise stated. *Studies by Waton *et al*. are annual UK National vascular registry reports.

### Thirty-day mortality

Fourteen of 26 studies reporting 30-day mortality were from North America^23,24,26,29–31,34,37,38,41–45^ (all from the USA). Six of 26 were from Europe and Central Asia^25,28,32,33,39,46^, and 2 were multicountry studies (1 using data only in HICs^11^ and 1 a global study^27^). Study populations ranged between 259^39^ and 186 338^41^.

All 26 studies were included in meta-analysis, with a mortality prediction interval of 4.0–20.4% (point estimate 9.4%, 95% c.i. 8.0 to 11.0%; *I*^2^ 99.47%, GRADE certainty analysis—very low, forest plot: *Fig. 5*).

**Figure 5:**
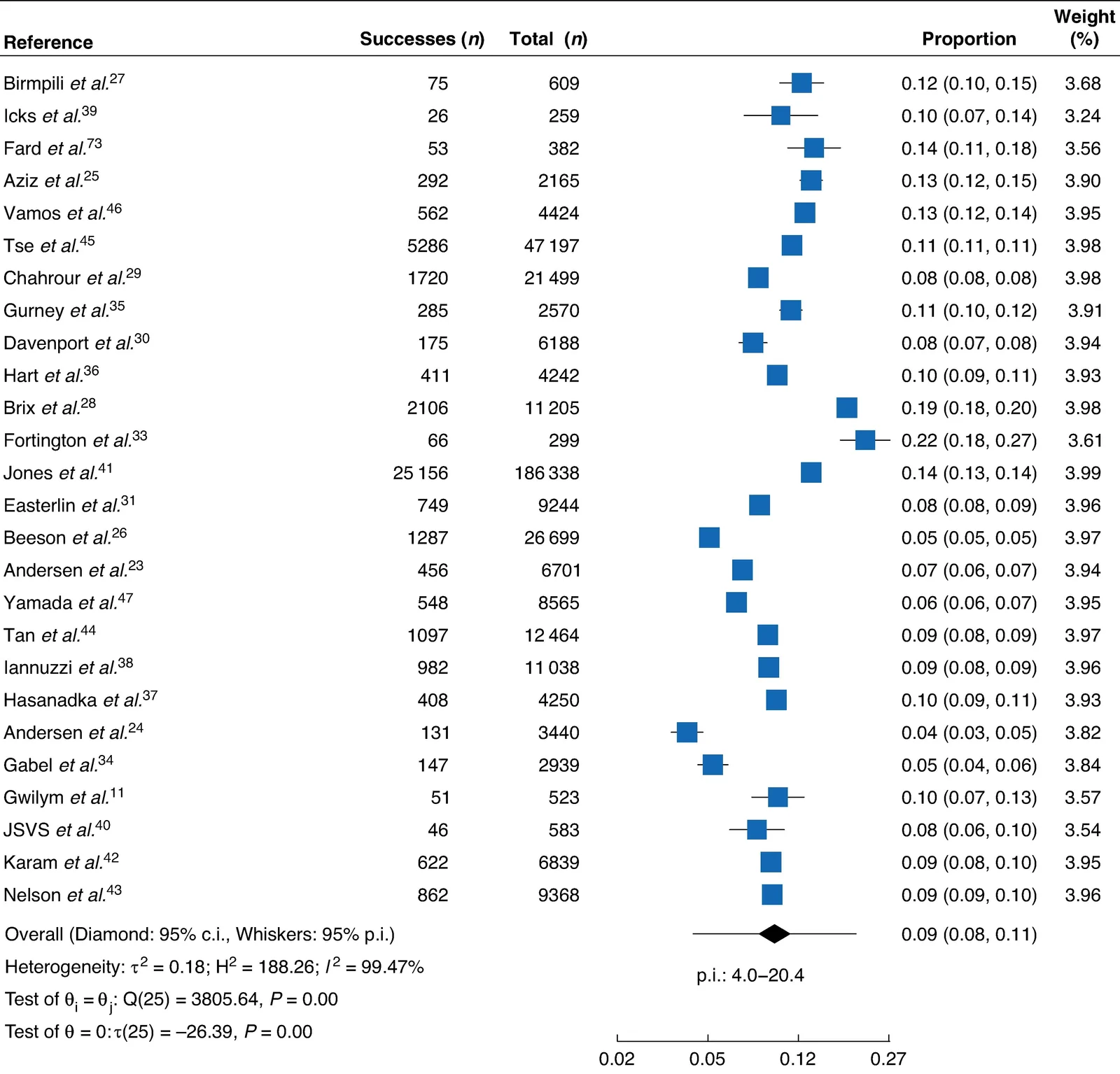
Thirty-day mortality after major amputation Whiskers around pooled estimate demonstrates 95% p.i. JSVS, Japanese Society for Vascular Surgery; c.i., confidence interval; p.i., prediction interval.

When all studies were examined together, there was no strong evidence of publication bias (Egger’s regression *P* = 0.107, funnel plot: *Fig. S8*). Leave-one-out analysis did not demonstrate a significant impact of any one individual study (*Fig. S9*).

Univariable meta-regression demonstrated that geographical region was significantly associated with mortality (24 studies: *P* = 0.016; *Fig. S10* (two multicountry studies were excluded from this analysis)). Studies from North America were significantly more likely to demonstrate a lower mortality level at 30 days compared with those from Europe and Central Asia (point estimates: North America 8%, 95% c.i. 6% to 10%; Europe and Central Asia 15%, 11% to 20%). To explore this, a region-specific test of publication bias was performed. Egger’s test demonstrated evidence of small study effects in studies from North America (Egger’s *P* = 0.001, funnel plot: *Fig. S11*). This suggests an over-representation of studies with lower mortality from this region. All other covariates (DM prevalence, mean population age, percentage of amputations that are TFA, year of publication, or country income status) did not demonstrate significance in univariable meta-regression (*Appendix S2*).

#### Sub-group analysis

Sixteen studies reported 30-day mortality after TTA^23–26,28–30,34,37,40,42,43,69–72^ (87 971), whilst 12 (42 145) reported outcomes after TFA^23–26,28–30,34,37,40,42,43^ (*Table 5*). The prediction interval of mortality after TFA was 4.0–26.6% (point estimate 10.9%, 95% c.i. 8.3% to 14.2%; *I*^2^ 98.71%, GRADE certainty of evidence—very low, forest plot: *Fig. 6*). For TTA, the prediction interval was 2.5–11.6% (point estimate 5.5%, 4.5% to 6.7%; *I*^2^ 97.28%, GRADE certainty of evidence—low, forest plot: *Fig. 7*). Small study effects were identified in 30-day outcomes after TFA (Egger’s regression *P* = 0.044, funnel plot: *Fig. S12*), but not in TTA outcomes (Egger’s regression *P* = 0.822, funnel plot: *Fig. S13*). Leave-one-out analysis did not demonstrate a significant impact of any one individual study (*Figs. S14*, *S15* for TTA and TFA, respectively).

**Figure 6:**
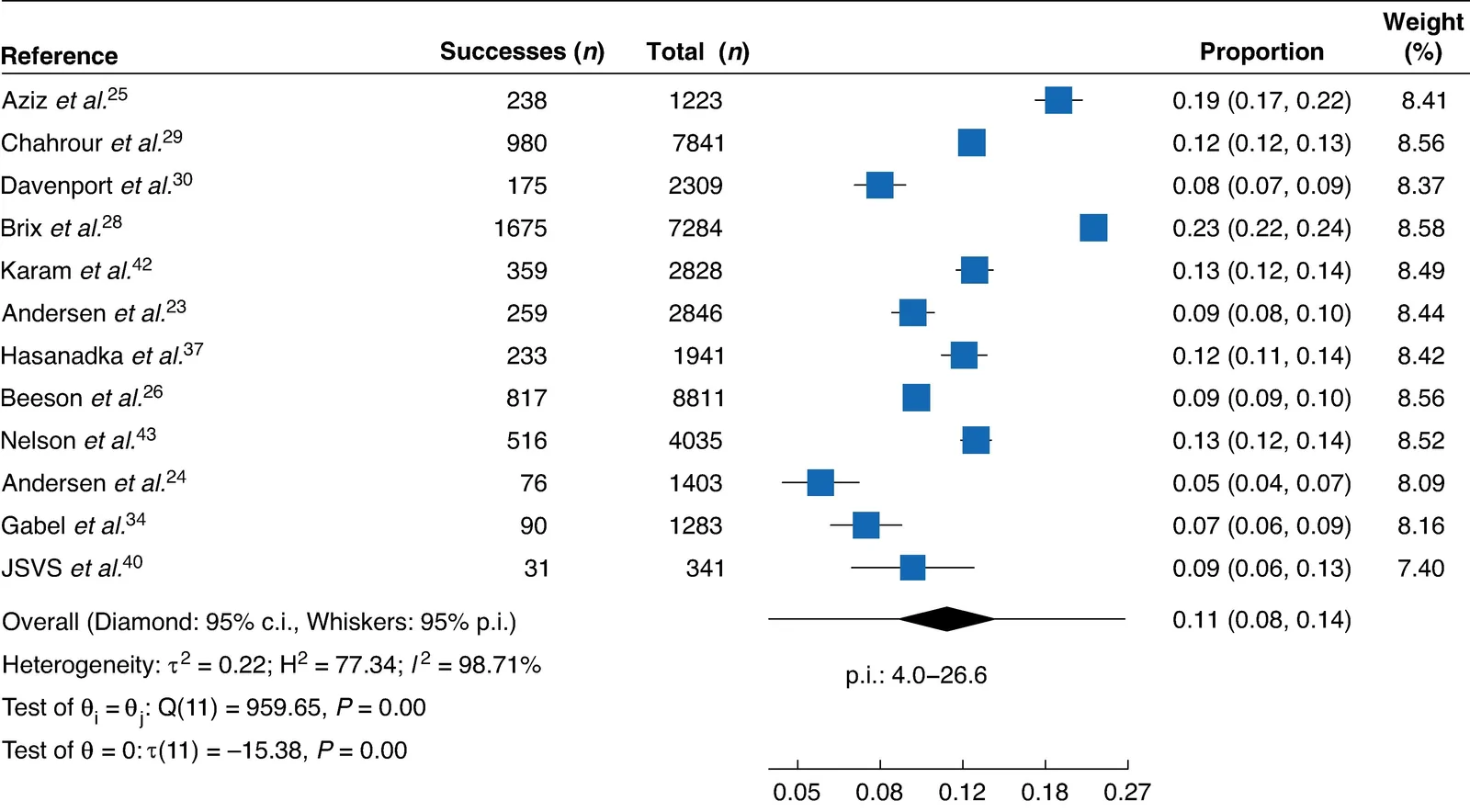
Thirty-day mortality after transfemoral amputation Whiskers around pooled estimate demonstrates 95% p.i. JSVS, Japanese Society for Vascular Surgery; c.i., confidence interval; p.i., prediction interval.

**Figure 7:**
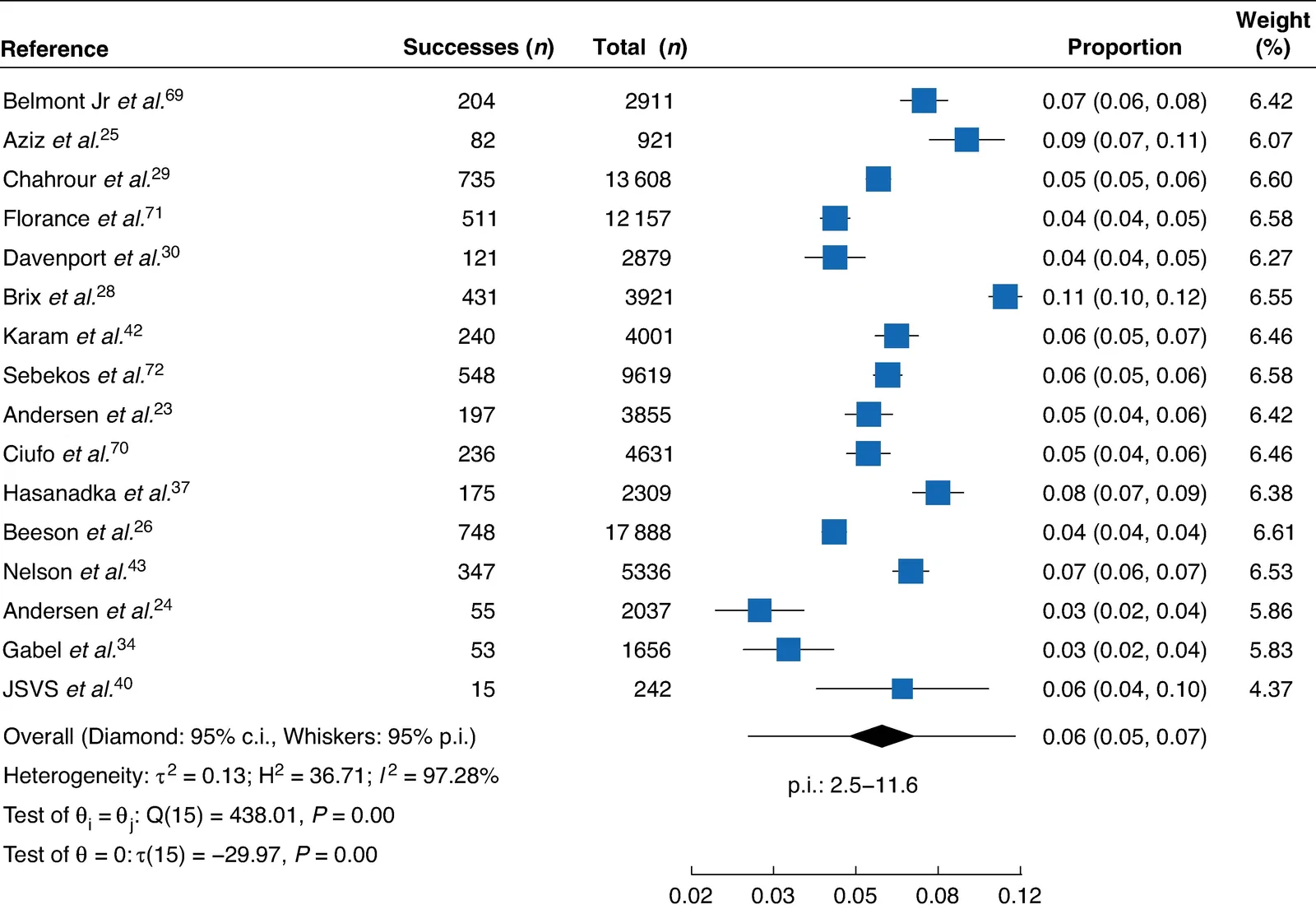
Thirty-day mortality after transtibial amputation Whiskers around pooled estimate demonstrates 95% p.i. JSVS, Japanese Society for Vascular Surgery; c.i., confidence interval; p.i., prediction interval.

**Table 5:** Transtibial and transfemoral amputation: 30-day mortality outcomes in studies that report them stratified by anatomical amputation level

Reference	Country (region)	TTA population (*n*)	TTA mortality (%)	TFA population (*n*)	TFA mortality (%)
Brix *et al.* 2024^28^	Denmark	3921	11.0	7284	23
Gabel *et al.* 2018^34^	USA	1656	3.2	1283	7.0
Andersen *et al.* 2021^24^	USA	2037	2.7	1403	5.4
Karam *et al.* 2013^42^	USA	4001	6..0	2828	12.7
Nelson *et al.* 2012^43^	USA	5336	6.5	4035	12.8
Andersen *et al.* 2019^23^	USA	3855	5.1	2846	9.1
Beeson *et al.* 2023^26^	USA (California)	17 888	4.2	8811	9.27
Hasanadka *et al.* 2011^37^	USA	2309	7.6	1941	12.0
Davenport *et al.* 2012^30^	USA	2879	4.2	2309	7.6
JSVS 2024^40^	Japan	242	6.2	341	9.0
Aziz *et al.* 2020^25^	Austria	921	8.9	1223	19.5
Chahrour *et al.* 2022^29^	USA	13 608	5.4	7841	12.5
Belmont Jr *et al.* 2011^69^	USA	2911	7.0		
Florance *et al.* 2023^71^	USA	12 157	4.2		
Ciufo *et al.* 2019^70^	USA	4631	5.1		
Sebekos *et al.* 2023^72^	USA	9619	5.7		

Values are *n* unless otherwise stated. Four studies (Belmont Jr *et al.* 2011^69^, Florance *et al.* 2023^71^, Cuifo *et al.* 2019^70^ and Sebekos *et al.* 2023^72^) reported only transtibial amputation 30-day mortality without reporting transfemoral amputation outcomes. JSVS, Japanese Society of Vascular Surgery; TTA, transtibial amputation; TFA, transfemoral amputation.

### Discussion

This review has demonstrated that the majority of studies reporting short-term outcomes after major lower limb amputation did so from high-income countries in Europe and North America. It has shown that up to one-in-four of those who undergo major lower limb amputations are at risk of in-hospital mortality, and up to one-in-five are at risk of 30-day mortality. However, it has also demonstrated significant variability and heterogeneity in reported mortality outcomes.

This substantial heterogeneity likely reflects genuine variability in mortality; however, the factors driving this variability could not be robustly detected. Geographical region was the only factor identified as contributing to variability, with significantly lower 30-day mortality estimates reported in studies from North America compared with Europe and Central Asia. However, small study effects in studies from this region complicate the interpretation of this finding.

Univariable meta-regression analysis did not detect an association between mortality and DM prevalence, year of study publication, mean age of study population, or the proportion of amputations that were TFAs. These factors were therefore not identified as sources of variability in the present findings, despite biological plausibility and previous evidence that suggests that they can impact outcomes^74^. Incomplete reporting is likely to contribute to the current finding of a lack of association, as 35% of studies did not report mean or median age, 25% did not report sex distribution, 31% did not report the prevalence of DM, and 31% did not report the percentage of TTAs *versus* TFAs. This limited the number of studies contributing to each meta-regression and precluded multivariable meta-regression. Thus, the literature is not adequately reported to support quantitative investigation of the impact of these moderating factors on outcomes. This deficiency has also been highlighted in previous reviews, which have suggested a more standardized approach to the reporting of population factors in this context^75^.

There are a number of other clinical and system-level factors that may vary across populations, and drive variability in outcomes amongst those who undergo amputation, but that were not investigated in this study. Clinical factors include the urgency of amputation and patient frailty, both of which have been shown to predict short-term mortality independently after major lower limb amputation^76,77^. The indication for amputation can also impact outcomes, with DM-related amputations associated with lower postoperative mortality compared with peripheral artery disease-related amputations^78^. The patients’ acute physiological state at presentation (for those undergoing non-elective amputation) is an additional factor associated with outcomes, with worse outcomes in those who present with severe presentations of sepsis^79^, as well as those who have chronic kidney disease, end-stage renal disease, congestive cardiac failure, and increased tissue loss in the affected limb^28,74^.

System-level factors also drive variability in outcomes, and therefore heterogeneity. For instance, socioeconomic status can impact whether amputation is performed as a primary procedure or after failed attempts at limb salvage. For example, patients in high-income countries often undergo repeated attempts at limb salvage before a decision is made to proceed to amputation^80^, whereas in many LMICs, a combination of resource limitations, particularly in relation to vascular surgery, and delays in patient presentation after the development of symptoms of an at-risk limb can result in more severe presentations and increased likelihood of primary amputation^62,81^. At a local level, variation in approach is likely to exist amongst centres within the same country and health system, and may also be related to socioeconomic status^58^. This may result in a different clinical profile of patients undergoing amputation, and therefore a differing postoperative mortality risk^82^.

Other system-level factors that can contribute to variability in outcomes include the availability and use of perioperative care pathways and risk stratification tools, affecting patient selection for surgery^83^. Differences in perioperative resource availability, including critical care facilities and enhanced recovery programmes, and differences in approach to palliation with an intact limb are additional factors. Finally, centre volume and specialist surgical service availability may impact outcomes^84^.

These system-level factors are rarely described in observational or registry studies, and their investigation may require prospective cohort studies that capture patient factors alongside system-level and pathway-level variables.

In addition, the heterogeneity in in-hospital mortality may also be impacted by length of hospital stay, which is not censored at 30 days in studies included in this review and may lead to higher estimates of in-hospital mortality.

Taken together, therefore, heterogeneity in this review may be partly attributable to region, but its source is also obscured by inconsistent reporting of basic population characteristics, study methodology, and unmeasured clinical and health-system differences.

Whilst this review excluded studies focused exclusively on narrow patient populations, such as those with end-stage renal failure or those who had attempted and failed limb-salvage vascular surgery, in including population-level administrative and registry data, such populations groups will still be represented in pooled estimates. Estimates reported in this review can therefore be interpreted as population-level average mortality estimates rather than estimates for specific high-risk groups. For example, patients with end-stage renal failure, acute sepsis, or congestive cardiac failure may have risks closer to the upper bound of the prediction intervals, whilst lower-risk elective patients are likely to have risks closer to the lower bound. As such, prediction intervals from this review can be used alongside individual patient factors and, where available, subgroup-specific prognostic evidence. Therefore, clinicians can use overall prediction intervals as a benchmark, then contextualize where an individual patient may sit within that range, according to their individual risk factors.

This highlights the benefit of prediction intervals. Although they are not routinely reported in systematic reviews, they can present the consequences of heterogeneity in a straightforward and clinically relevant way, using the same scale as the original study, in this case mortality after major amputation^85,86^. Whilst confidence intervals and prediction intervals coincide when between-study heterogeneity is low, prediction intervals allow a clinically meaningful conclusion to be drawn when heterogeneity is high^87^. They can also be used to estimate what 95% of the true effects will be in future studies, facilitating power calculations for researchers^88^.

The finding that 30-day mortality is lower in studies published in North America, in addition to small study effects in these studies, suggests the possibility of publication bias. This, along with the findings suggestive of publication bias for in-hospital outcomes (in TTAs and TFAs), strengthens the argument for standardized registry-based reporting of outcomes after major amputations, to allow researchers and clinicians to determine the differences in populations who may be undergoing amputation in different regions. It is, however, also important to consider that assessments using Egger’s meta-regression and funnel plot asymmetry can be influenced by genuine heterogeneity in study populations and methodological differences between large and small studies^89^.

Geography, and the clinical context within this, may be a significant factor in explaining variability in outcomes after amputation, but current geographical spread of studies capturing data on outcomes after amputation is limited. Most studies included in this review are from Europe and the USA, with only three from LMICs. This is despite an intentionally wide search strategy to identify studies from LMICs. This demonstrates that, despite efforts by the Lancet Commission on Global surgery to encourage research into surgical outcomes in LMICs, data remain sparse when it comes to major amputations^15^. Whilst major amputations are not a bellwether procedure as designated by the Lancet Commission, it is the authors’ view that understanding amputations informs researchers and policymakers of the underlying functioning of a health system. This is because amputations cut across the vertical health-systems approach and can reflect multiple aspects of a health-systems performance, including effectiveness of long-term management of diabetes and cardiovascular diseases, trauma and orthopaedic care capacity, access to timely surgical intervention, and the burden of infections, particularly after surgery, and resultant mortality. Therefore, planned research into the outcomes of those who undergo amputation in low- and middle-income countries is urgently required^90^.

Whilst the range of mortality outcomes reported in this review is consistent with a previously published review^75^, point estimates in this review were higher than some^91^. Methodological differences including reliance on mean mortality, weighting, inclusion of studies not limited to those done at a population level, and time frames for included studies likely explain differences.

It could be considered that a limitation of this study is that, in aiming to minimize bias and heterogeneity, it only included population-based studies. This may have resulted in reduced quality of evidence being obtained, particularly from LMICs. However, including only population-based studies limited the impact of individual hospitals’ selection bias impacting prediction intervals. Another limitation is the reliance on reports sourced from the same database. For instance, seven of the studies reporting in-hospital mortality originated from the UK National Vascular Registry, which may contribute to geographic bias. Whilst such registries utilize routinely collected data, which means they can provide an understanding of real-world outcomes, limitations include a possible lack of granularity of data, missing data, and ascertainment bias, particularly in voluntary registries. In addition, the lack of uniform reporting of data on covariables such as comorbidity precluded multivariable meta-regression. Whilst univariable meta-regression was performed, results from this cannot be interpreted as independent effects, and causal inference on the relative contribution of each factor cannot be ascertained from this study.

The main limitation of this study, however, is the heterogeneity, which limits the use of point estimates from meta-analyses and contributed to low or very-low GRADE certainty assessments. Nevertheless, this was mitigated by primarily reporting prediction intervals rather than just point estimates, therefore providing a clinically relevant outcome that can also be used by researchers who wish to investigate further major amputation outcomes. Overlapping study populations are also a limitation of this study. Whilst no studies used identical data over identical time periods, there was partial overlap in study populations, which may bias pooled estimates.

Early postoperative mortality after major lower limb amputation remains substantial, though the risk can vary in magnitude. The factors that contribute to this variability are not clear. Whilst there may be variation by geographical context, this requires further investigation, as does the impact of repeated limb-salvage attempts followed by amputation *versus* primary amputation and the extent of comorbidities. Understanding these outcomes will depend on future research reporting uniform patient- and system-level factors that can influence amputation outcome.

## Supplementary Material

zrag122_Supplementary_Data

## Data Availability

Data available upon request.
