# Supplementary material for: Short-term outcomes after major lower limb amputation: meta-analysis
Source: BJS Open. 2026 Oct 23;10(5):zrag122. doi: 10.1093/bjsopen/zrag122 (PMC13692688; doi:10.1093/bjsopen/zrag122)
Supplement: Supplementary file 1 [file zrag122_supplementary_data.docx]

Short-term outcomes after major lower limb amputation: meta-analysis

Nick Moody,^1^ Rhiannon Nielsen,^2^ Megan Lyons,^3^ Leanne Murphy,^4^ Becky Sandford,^5,6^ David C Bosanquet,^7,8^ Kathryn Chu,^9^ Matthew Popplewell,^1^ Justine Davies^1^

^1^Department of Applied Health Sciences, College of Medicine and Health, University of Birmingham, Birmingham, UK

^2^ Gwent Vascular Institute, Royal Gwent Hospital, South East Wales Vascular Network, UK

^3^ Imperial College Hospitals NHS trust

^4^ Lewisham and Greenwich NHS trust

^5^ Guy’s and St Thomas’ NHS Trust, London, UK

^6^ King’s College London, London, UK

^7^ South East Wales Vascular Network, University Hospital of Wales, Cardiff, Wales

^8^ School of Medicine, Cardiff University, Cardiff, UK

^9^ Centre for Global Surgery, Stellenbosch University Faculty of Medicine and Health Sciences, Cape Town, South Africa

**Corresponding author.** Nick Moody, Department of Applied Health Sciences, College of Medicine and Health, University of Birmingham, Birmingham, UK

**ORCID ID**; 0000-0002-6369-8851. **Twitter: Linkedin:** [www.linkedin.com/in/nick-moody-706b93325](http://www.linkedin.com/in/nick-moody-706b93325)

Table of Contents

[Supplementary tables	4](#_Toc229396310)

[Supplementary table S1: PRISMA checklist	4](#_Toc229396311)

[Supplementary table S2: Search strategy	7](#_Toc229396312)

[Supplementary table S3: PICO table	9](#_Toc229396313)

[Supplementary table S4: Newcastle-Ottowa quality assessment of included studies	10](#_Toc229396314)

[Supplementary table S5: detailed GRADE assessment	14](#_Toc229396315)

[Supplementary figures	16](#_Toc229396316)

[Supplementary figure S1 leave one out analysis, in hospital mortality	16](#_Toc229396317)

[Supplementary figure S2: univariable meta-regression in-hospital mortality:geographical region	17](#_Toc229396318)

[Supplementary figure S3: Funnel plot, in-hospital mortality	18](#_Toc229396319)

[Supplementary figure S4: Funnel plot, In-hospital mortality, transtibial amputations	18](#_Toc229396320)

[Supplementary figure S5: Funnel plot, in-hospital mortality, transfemoral amputations	19](#_Toc229396321)

[Supplementary figure S6: Leave one out analysis, transtibial amputations. In-hospital mortality	19](#_Toc229396322)

[Supplementary figure S7: leave one out analysis, Transfemoral amputations, in hospital mortality	20](#_Toc229396323)

[Supplementary figure S8: funnel plot, 30-day mortality	20](#_Toc229396324)

[Supplementary figure S9: leave-one out analysis, 30-day mortality	21](#_Toc229396325)

[Supplementary figure S10: univariable meta-regression, 30-day mortality, geographical region	22](#_Toc229396326)

[Supplementary figure S11: funnel plot, 30-day mortality, North America region	23](#_Toc229396327)

[Supplementary figure S12: Funnel plot. 30-day mortality, transfemoral amputations.	23](#_Toc229396328)

[Supplementary figure S13: Funnel plot. 30-day mortality, transtibial amputations.	24](#_Toc229396329)

[Supplementary figure S14: leave one out analysis. 30-day mortality, transtibial amputations	24](#_Toc229396330)

[Supplementary figure S15: leave one out analysis, 30-day mortality, transfemoral amputations	26](#_Toc229396331)

[Supplementary Appendixes	26](#_Toc229396332)

[Appendix 1 Univariable meta-regression, In-hospital mortality	26](#_Toc229396333)

[Age	27](#_Toc229396334)

[Year of publication	28](#_Toc229396335)

[Diabetes prevalence	29](#_Toc229396336)

[Income level of study	30](#_Toc229396337)

[World bank geographical region of study	31](#_Toc229396338)

[percentage TFA	32](#_Toc229396339)

[Appendix 2: Univariable meta-regression, 30-day mortality	32](#_Toc229396340)

[Year of publication	32](#_Toc229396341)

[Diabetes prevalence	34](#_Toc229396342)

[World bank geographical region of study	35](#_Toc229396343)

[Age	36](#_Toc229396344)

[Percentage TFA	36](#_Toc229396345)

# Supplementary tables

## Supplementary table S1: PRISMA checklist

| **Section and Topic** | **Item #** | **Checklist item** | **Location where item is reported** |
| --- | --- | --- | --- |
| **TITLE** | | |  |
| Title | 1 | Identify the report as a systematic review. | Title |
| **ABSTRACT** | | |  |
| Abstract | 2 | See the PRISMA 2020 for Abstracts checklist. |  |
| **INTRODUCTION** | | |  |
| Rationale | 3 | Describe the rationale for the review in the context of existing knowledge. | Page 4 |
| Objectives | 4 | Provide an explicit statement of the objective(s) or question(s) the review addresses. | Page 4 |
| **METHODS** | | |  |
| Eligibility criteria | 5 | Specify the inclusion and exclusion criteria for the review and how studies were grouped for the syntheses. | Page 5 -6 |
| Information sources | 6 | Specify all databases, registers, websites, organisations, reference lists and other sources searched or consulted to identify studies. Specify the date when each source was last searched or consulted. | Page 5 |
| Search strategy | 7 | Present the full search strategies for all databases, registers and websites, including any filters and limits used. | Supplemental appendix S2 |
| Selection process | 8 | Specify the methods used to decide whether a study met the inclusion criteria of the review, including how many reviewers screened each record and each report retrieved, whether they worked independently, and if applicable, details of automation tools used in the process. | Page 5 - 6 |
| Data collection process | 9 | Specify the methods used to collect data from reports, including how many reviewers collected data from each report, whether they worked independently, any processes for obtaining or confirming data from study investigators, and if applicable, details of automation tools used in the process. | Page 6 |
| Data items | 10a | List and define all outcomes for which data were sought. Specify whether all results that were compatible with each outcome domain in each study were sought (e.g. for all measures, time points, analyses), and if not, the methods used to decide which results to collect. | Page 6 |
|  | 10b | List and define all other variables for which data were sought (e.g. participant and intervention characteristics, funding sources). Describe any assumptions made about any missing or unclear information. | Page 6 |
| Study risk of bias assessment | 11 | Specify the methods used to assess risk of bias in the included studies, including details of the tool(s) used, how many reviewers assessed each study and whether they worked independently, and if applicable, details of automation tools used in the process. | Page 6 |
| Effect measures | 12 | Specify for each outcome the effect measure(s) (e.g. risk ratio, mean difference) used in the synthesis or presentation of results. | Page 7 |
| Synthesis methods | 13a | Describe the processes used to decide which studies were eligible for each synthesis (e.g. tabulating the study intervention characteristics and comparing against the planned groups for each synthesis (item #5)). | Page 5-6 |
|  | 13b | Describe any methods required to prepare the data for presentation or synthesis, such as handling of missing summary statistics, or data conversions. | Page 6-7 |
|  | 13c | Describe any methods used to tabulate or visually display results of individual studies and syntheses. | Page 6-7 |
|  | 13d | Describe any methods used to synthesize results and provide a rationale for the choice(s). If meta-analysis was performed, describe the model(s), method(s) to identify the presence and extent of statistical heterogeneity, and software package(s) used. | Page 7 |
|  | 13e | Describe any methods used to explore possible causes of heterogeneity among study results (e.g. subgroup analysis, meta-regression). | Page 7 |
|  | 13f | Describe any sensitivity analyses conducted to assess robustness of the synthesized results. | Page 7 |
| Reporting bias assessment | 14 | Describe any methods used to assess risk of bias due to missing results in a synthesis (arising from reporting biases). | Page 7 |
| Certainty assessment | 15 | Describe any methods used to assess certainty (or confidence) in the body of evidence for an outcome. | Page 6 - 7 |
| **RESULTS** | | |  |
| Study selection | 16a | Describe the results of the search and selection process, from the number of records identified in the search to the number of studies included in the review, ideally using a flow diagram. | Page 8 |
|  | 16b | Cite studies that might appear to meet the inclusion criteria, but which were excluded, and explain why they were excluded. | Page 8 |
| Study characteristics | 17 | Cite each included study and present its characteristics. | Page 16 - 18 |
| Risk of bias in studies | 18 | Present assessments of risk of bias for each included study. | Supplemental appendix S |
| Results of individual studies | 19 | For all outcomes, present, for each study: (a) summary statistics for each group (where appropriate) and (b) an effect estimate and its precision (e.g. confidence/credible interval), ideally using structured tables or plots. | Page 16 - 18 |
| Results of syntheses | 20a | For each synthesis, briefly summarise the characteristics and risk of bias among contributing studies. | Page 9 - 11 |
|  | 20b | Present results of all statistical syntheses conducted. If meta-analysis was done, present for each the summary estimate and its precision (e.g. confidence/credible interval) and measures of statistical heterogeneity. If comparing groups, describe the direction of the effect. | Page 9 -11 |
|  | 20c | Present results of all investigations of possible causes of heterogeneity among study results. | Page 9 - 11 |
|  | 20d | Present results of all sensitivity analyses conducted to assess the robustness of the synthesized results. | Page 9 -11 |
| Reporting biases | 21 | Present assessments of risk of bias due to missing results (arising from reporting biases) for each synthesis assessed. | Page 9 -11 |
| Certainty of evidence | 22 | Present assessments of certainty (or confidence) in the body of evidence for each outcome assessed. | Page 9 -11 |
| **DISCUSSION** | | |  |
| Discussion | 23a | Provide a general interpretation of the results in the context of other evidence. | Page 12 |
|  | 23b | Discuss any limitations of the evidence included in the review. | Page 14 |
|  | 23c | Discuss any limitations of the review processes used. | Page 14 |
|  | 23d | Discuss implications of the results for practice, policy, and future research. | Page 12 - 14 |
| **OTHER INFORMATION** | | |  |
| Registration and protocol | 24a | Provide registration information for the review, including register name and registration number, or state that the review was not registered. | Page 5 |
|  | 24b | Indicate where the review protocol can be accessed, or state that a protocol was not prepared. | Page 5 |
|  | 24c | Describe and explain any amendments to information provided at registration or in the protocol. | - |
| Support | 25 | Describe sources of financial or non-financial support for the review, and the role of the funders or sponsors in the review. | End |
| Competing interests | 26 | Declare any competing interests of review authors. | End |
| Availability of data, code and other materials | 27 | Report which of the following are publicly available and where they can be found: template data collection forms; data extracted from included studies; data used for all analyses; analytic code; any other materials used in the review. | End |

## Supplementary table S2: Search strategy

#### 1 MEDLINE

Database: MEDLINE (PubMed, NLM)

Search period: unlimited

Date of search:

| **Step** | **Hits** | **Search** |
| --- | --- | --- |
| #1 | 24,938 | "AMPUTATION"[MESH] |
| #2 | 55,027 | disarticulation [tiab] OR amputat* [tiab] OR amputee [tiab] OR Limb amputation [tiab] OR lower extremity amputation [tiab] OR limb loss [tiab] OR lower limb loss [tiab] |
| #3 | 61,832 | #1 OR #2 |
| #4 | 28,668 | "EPIDEMIOLOGY"[MESH] |
| #5 | 745,727 | epidemiolog* [ti] OR prevalence [ti] OR incidence [ti] OR frequency [ti] OR population survey [tiab] OR survey data [tiab] OR administrat* data [tiab] OR occurence [ti] OR population data [tiab] OR population-based [tiab] |
| #6 | 762,194 | #4 OR #5 |
| #7 | 760,147 | Aetiolog*(ti) OR Cause (ti) OR outcome* (ti) OR Mortality (ti) OR death (ti) OR complication* (ti) OR morbidity |
| #8 | 1,486,235 | #6 OR #7 |
| #9 | 4782 | #3 AND #8 |
| #10 | 3106 | Limit 2008 - 2024 |

SCOPUS search strategy

| **Step** | **Hits** | **Search** |
| --- | --- | --- |
| #1 | 123, 457 | ( TITLE-ABS-KEY ( amputation ) OR TITLE-ABS-KEY ( disarticulation ) OR TITLE-ABS-KEY ( amputat* ) OR TITLE-ABS-KEY ( amputee ) OR TITLE-ABS-KEY ( limb AND amputat* ) OR TITLE-ABS-KEY ( lower AND extremity AND amputat* ) OR TITLE-ABS-KEY ( limb AND loss ) OR TITLE-ABS-KEY ( lower AND limb AND loss ) ) |
| #2 | 651, 282 | KEY ( epidemiology ) |
| #3 | 1,105,026 | ( TITLE ( epidemiology ) OR TITLE ( prevalence ) OR TITLE ( incidence ) OR TITLE ( frequency ) OR TITLE ( population AND survey ) OR TITLE ( survey AND data ) OR TITLE ( administrative AND data ) OR TITLE ( occurence ) OR TITLE ( population AND data ) OR TITLE ( population AND based ) ) |
| #4 | 1,656,745 | #2 OR #3 |
| #5 | 1,438,511 | ( TITLE ( aetiolog* ) OR TITLE ( cause ) OR TITLE ( outcome* ) OR TITLE ( mortality ) OR TITLE ( death ) OR TITLE ( complication* ) OR TITLE ( morbidity ) ) |
| #6 | 2,991,026 | #4 OR #5 |
| #7 | 11,873 | #1 AND #6 |
| #8 | 8,959 | Limit 2008 - 2024 |
| #9 |  |  |
|  |  |  |

#### 2 EMBASE, Journals@OVID

Database: MEDLINE (1946-2012), EMBASE (1974-2012), Journals@OVID (OVID)

Search period: unlimited

| **Step** | **Hits** | **Search** |
| --- | --- | --- |
| #1 | 90,601 | (amputation or disarticulation or amputat* or amputee or Limb amputation or lower extremity amputation or limb loss or lower limb loss).mp. [mp=title, abstract, heading word, drug trade name, original title, device manufacturer, drug manufacturer, device trade name, keyword heading word, floating subheading word, candidate term word] |
| #2 | 686,907 | (epidemiology or prevalence or incidence or frequency or population survey or survey data or survey data or population based).m_titl. |
| #3 | 853,829 | (aetiolog* or cause or outcome or mortality or death or complication or morbidity).m_titl. |
| #4 | 151,2155 | #2 OR #3 |
| #5 | 5487 | #1 AND #4 |
| #6 | 4184 | Limit 2008 - 2024 |

## Supplementary table S3: PICO table

|  | Inclusion Criteria | Exclusion Criteria |
| --- | --- | --- |
| Population | Populations who undergo major lower limb amputations for any aetiology  Population level studies: those across broad and well defined regional or national populations | Studies limited to pre-specified high risk populations, i.e. those on dialysis, or who have undergone vascular intervention  Studies which report incidence in war settings  Single centre studies  Studies confined to war/conflict settings |
| Intervention | MLLA, defined as amputation at or proximal to ankle joint (studies which report MLLA but do not define it are also included) | Minor amputations (distal to ankle joint)  Studies which pool minor and major amputation outcomes without specifying for major amputations separately |
| Comparison | NA | NA |
| Outcome | Studies which report in-hospital mortality outcomes as a primary or secondary outcome  Studies which report 30-day mortality outcomes as a primary or secondary outcome | Studies which do not provide a 30-day or in-hospital mortality outcome after major amputation  Studies which provide only longer term mortality outcomes |
| Type of publication | Quantitative or mixed-methods studies published in peer-reviewed journals. Reports from health organisations.  Grey literature.  Registry reports | Qualitative studies  Case reports  Descriptive reviews  Letters or opinion based articles |
| Timeframe | published between 01^st^ January 2008 and 30^th^ November 2025. | published prior to 2008 |
| Language | published in English, French, Spanish, German or Portuguese languages only. | Publication not in English, French, Spanish, German or Portuguese languages. |

## **Supplementary table S4**: Newcastle-Ottowa quality assessment of included studies

|  | Representativeness of the exposed cohort | Selection of non-exposed cohort | Ascertainment of exposure | outcome of interest not present at start of study | Study controls for 1 factor | Study controls for 2 factors | Assessment of outcome | follow up long enough | Was follow up of cohorts adequate | Total |
| --- | --- | --- | --- | --- | --- | --- | --- | --- | --- | --- |
| Birmpili 2024* | 1 | 1 | 1 | 1 | 1 | 0 | 0 | 1 | 1 | 7 |
| Portela 2024 | 1 | 1 | 1 | 1 | 1 | 0 | 0 | 1 | 1 | 7 |
| Moxey 2012 | 1 | 1 | 1 | 1 | 1 | 0 | 1 | 1 | 1 | 8 |
| Moxey 2010 | 1 | 1 | 1 | 1 | 0 | 0 | 1 | 1 | 1 | 7 |
| Endoh 2017 | 1 | 1 | 1 | 1 | 0 | 0 | 1 | 1 | 1 | 7 |
| Easterlin 2013 | 1 | 1 | 1 | 1 | 0 | 0 | 1 | 1 | 1 | 7 |
| Tan 2015 | 1 | 1 | 1 | 1 | 0 | 0 | 1 | 1 | 1 | 7 |
| Iannuzzi 2013 | 1 | 1 | 1 | 1 | 0 | 0 | 1 | 1 | 1 | 7 |
| Yamada 2016 | 1 | 1 | 1 | 1 | 0 | 0 | 1 | 1 | 1 | 7 |
| Brix 2024 | 1 | 1 | 1 | 1 | 0 | 0 | 1 | 1 | 1 | 7 |
| Gabel - 2018 | 1 | 1 | 1 | 1 | 0 | 0 | 1 | 1 | 1 | 7 |
| Andersen – 2020 | 1 | 1 | 1 | 1 | 0 | 0 | 1 | 1 | 1 | 7 |
| Karam 2013 | 1 | 1 | 1 | 1 | 0 | 0 | 1 | 1 | 1 | 7 |
| Nelson 2012 | 1 | 1 | 1 | 1 | 0 | 0 | 1 | 1 | 1 | 7 |
| BelmontJr 2011 | 1 | 1 | 1 | 1 | 0 | 0 | 1 | 1 | 1 | 7 |
| Andersen 2020 | 1 | 1 | 1 | 1 | 0 | 0 | 1 | 1 | 1 | 7 |
| Florance 2023 | 1 | 1 | 1 | 1 | 0 | 0 | 1 | 1 | 1 | 7 |
| Ciufo 2019 | 1 | 1 | 1 | 1 | 0 | 0 | 1 | 1 | 1 | 7 |
| Sebekos 2023 | 1 | 1 | 1 | 1 | 0 | 0 | 1 | 1 | 1 | 7 |
| Fortington 2013 | 1 | 1 | 1 | 1 | 0 | 0 | 1 | 1 | 1 | 7 |
| Hart 2024 | 1 | 1 | 1 | 1 | 0 | 0 | 1 | 1 | 1 | 7 |
| Andersen 2019 | 1 | 1 | 1 | 1 | 0 | 0 | 1 | 1 | 1 | 7 |
| Feinglass 2008 | 1 | 1 | 0 | 1 | 0 | 0 | 1 | 1 | 1 | 6 |
| Fard 2020 | 1 | 1 | 1 | 1 | 1 | 0 | 1 | 1 | 1 | 8 |
| Beeson 2023 | 1 | 1 | 1 | 1 | 0 | 0 | 1 | 1 | 1 | 7 |
| Gwilym - 2022 | 1 | 1 | 1 | 1 | 0 | 0 | 1 | 1 | 1 | 7 |
| Humphries 2016 | 1 | 1 | 1 | 1 | 0 | 0 | 1 | 1 | 1 | 7 |
| Tchankoni 2024 | 1 | 1 | 1 | 1 | 0 | 0 | 1 | 1 | 0 | 6 |
| Tse 2021 | 0 | 1 | 1 | 1 | 0 | 0 | 1 | 1 | 1 | 6 |
| Malyar 2013 | 0 | 1 | 1 | 1 | 0 | 0 | 1 | 1 | 1 | 6 |
| Vamos 2010 | 1 | 1 | 1 | 1 | 0 | 0 | 1 | 1 | 1 | 7 |
| Long 2020 | 1 | 1 | 1 | 1 | 0 | 0 | 1 | 1 | 1 | 7 |
| Davenport 2012 | 1 | 1 | 1 | 1 | 0 | 0 | 1 | 1 | 1 | 7 |
| DiGiovanni 2021 | 1 | 1 | 1 | 1 | 0 | 0 | 1 | 1 | 1 | 7 |
| Aziz 2020 | 1 | 1 | 1 | 1 | 0 | 0 | 1 | 1 | 1 | 7 |
| Gurney 2018 | 1 | 1 | 1 | 1 | 0 | 0 | 1 | 1 | 1 | 7 |
| Lopez-de-Andres 2016 | 1 | 1 | 1 | 1 | 0 | 0 | 1 | 1 | 1 | 7 |
| Icks 2011 | 0 | 1 | 1 | 1 | 0 | 0 | 1 | 1 | 1 | 6 |
| Lopez-de-Andres 2015 | 1 | 1 | 1 | 1 | 0 | 0 | 1 | 1 | 1 | 7 |
| SchuylerJones 2013 | 0 | 1 | 1 | 1 | 0 | 0 | 1 | 1 | 0 | 5 |
| Lim 2018 | 0 | 1 | 1 | 1 | 0 | 0 | 1 | 1 | 1 | 6 |
| JVS 2024 | 1 | 1 | 1 | 1 | 0 | 0 | 1 | 1 | 1 | 7 |
| Low 2017 | 0 | 1 | 1 | 1 | 0 | 0 | 1 | 1 | 1 | 6 |
| CIHI | 1 | 1 | 1 | 1 | 0 | 0 | 1 | 1 | 1 | 7 |
| Waton 2024 | 1 | 1 | 1 | 1 | 0 | 0 | 1 | 1 | 1 | 7 |
| Waton 2025 | 1 | 1 | 1 | 1 | 0 | 0 | 1 | 1 | 1 | 7 |
| Waton 2023 | 1 | 1 | 1 | 1 | 0 | 0 | 1 | 1 | 1 | 7 |
| Waton 2022 | 1 | 1 | 1 | 1 | 0 | 0 | 1 | 1 | 1 | 7 |
| Waton 2021 | 1 | 1 | 1 | 1 | 0 | 0 | 1 | 1 | 1 | 7 |
| Waton 2019 | 1 | 1 | 1 | 1 | 0 | 0 | 1 | 1 | 1 | 7 |
| Waton 2016 | 1 | 1 | 1 | 1 | 0 | 0 | 1 | 1 | 1 | 7 |
| Maheswaran 2024 | 1 | 1 | 1 | 1 | 0 | 0 | 1 | 1 | 1 | 7 |
| Hasanadka 2011 | 1 | 1 | 1 | 1 | 0 | 0 | 1 | 1 | 1 | 7 |
| Chahrour 2022 | 1 | 1 | 1 | 1 | 0 | 0 | 1 | 1 | 1 | 7 |

## Supplementary table S5: detailed GRADE assessment

| **№ of studies** | **Certainty assessment** | | | | | | **Effect** | | | **Certainty** | **Importance** |
| --- | --- | --- | --- | --- | --- | --- | --- | --- | --- | --- | --- |
|  | **Study design** | **Risk of bias** | **Inconsistency** | **Indirectness** | **Imprecision** | **Other considerations** | **№ of events** | **№ of individuals** | **Rate (95% CI)** |  |  |
| In-hospital mortality | | | | | | | | | | | |
| 25 | non-randomised studies | serious | very serious | not serious | serious | strong association dose response gradient |  |  |  | ⨁⨁◯◯ Low |  |
| 30-day mortality | | | | | | | | | | | |
| 26 | non-randomised studies | very serious | very serious | not serious | serious | publication bias strongly suspected all plausible residual confounding would suggest spurious effect, while no effect was observed dose response gradient |  |  |  | ⨁◯◯◯ Very low |  |
| In-hospital TFA mortality | | | | | | | | | | | |
|  | non-randomised studies | serious | very serious | not serious | serious | publication bias strongly suspected strong association dose response gradient |  |  |  | ⨁◯◯◯ Very low |  |
| In-hospital TTA mortality | | | | | | | | | | | |
|  | non-randomised studies | serious | very serious | not serious | serious | publication bias strongly suspected strong association dose response gradient |  |  |  | ⨁◯◯◯ Very low |  |
| 30-day TFA mortality | | | | | | | | | | | |
|  | non-randomised studies | very serious | serious | not serious | serious | publication bias strongly suspected strong association dose response gradient |  |  |  | ⨁◯◯◯ Very low |  |
| 30-day TTA mortality | | | | | | | | | | | |
|  | non-randomised studies | serious | very serious | not serious | serious | strong association dose response gradient |  |  |  | ⨁⨁◯◯ Low |  |

# Supplementary figures

## **Supplementary figure S1** leave one out analysis, in hospital mortality

## **Supplementary figure S2:** univariable meta-regression in-hospital mortality:geographical region

Figure: In-hospital meta-regression geography – not significant.

## Supplementary figure S3: Funnel plot, in-hospital mortality

## Supplementary figure S4: Funnel plot, In-hospital mortality, transtibial amputations

## Supplementary figure S5: Funnel plot, in-hospital mortality, transfemoral amputations

## Supplementary figure S6: Leave one out analysis, transtibial amputations. In-hospital mortality

## Supplementary figure S7: leave one out analysis, Transfemoral amputations, in hospital mortality

## Supplementary figure S8: funnel plot, 30-day mortality

## Supplementary figure S9: leave-one out analysis, 30-day mortality

## Supplementary figure S10: univariable meta-regression, 30-day mortality, geographical region

## Supplementary figure S11: funnel plot, 30-day mortality, North America region

## Supplementary figure S12: Funnel plot. 30-day mortality, transfemoral amputations.

## Supplementary figure S13: Funnel plot. 30-day mortality, transtibial amputations.

## Supplementary figure 14: leave one out analysis. 30-day mortality, transtibial amputations

## Supplementary figure 15: leave one out analysis, 30-day mortality, transfemoral amputations

# Supplementary Appendixes

## Appendix 1 Univariable meta-regression, In-hospital mortality

### Age

**In-hospital mortality univariable regression:**

### Year of publication

### Diabetes prevalence

### Income level of study

### World bank geographical region of study

### percentage TFA

## Appendix 2: Univariable meta-regression, 30-day mortality

**Univariable regression**

### Year of publication

### Diabetes prevalence

### World bank geographical region of study

**(1= Europe, 2= North America, 3= east Asia and pacific,****)**

### Age

### Percentage TFA

**Supplementary Figures and Tables**

**References**
